# Comparative Evaluation of Two Modified Glass Ionomer Cements with Conventional Glass Ionomer Cement for Fracture Toughness using 3-point Bending Test

**DOI:** 10.21142/2523-2754-1403-2026-297

**Published:** 2026-07-10

**Authors:** Trisha Patel, Farhin Katge, Devendra Patil, Vamsi Krishna, Parin Bhanushali

**Affiliations:** 1 Department of Pediatric and Preventive Dentistry, Terna Dental College. Navi-Mumbai, Maharashtra, India. patelt26@gmail.com, dev1987endra@gmail.com, kc.vamsi@gmail.com, dr.parin91@hotmail.com Department of Pediatric and Preventive Dentistry Terna Dental College Navi-Mumbai Maharashtra India patelt26@gmail.com dev1987endra@gmail.com kc.vamsi@gmail.com dr.parin91@hotmail.com

**Keywords:** Glass Ionomer Cement, Fracture Toughness, 3-point Bending Test, Artificial Saliva, cemento de ionómero de vidrio, tenacidad a la fractura, ensayo de flexión de 3 puntos, saliva artificial

## Abstract

**Objective:**

The purpose of this study was to evaluate and compare the fracture toughness of Advanced glass hybrid Glass Ionomer Cement (GIC), Zirconium reinforced GIC and conventional GIC using 3-point bending test.

**Materials and Methods:**

Three groups were formed in the present study, Group 1 (Hybrid GIC), Group 2 (Zirconomer Improved), Group 3 (Conventional GIC) which were tested at 24 hours and 7 days time interval according to their storage time in artificial saliva. Samples of each material were prepared with the help of customized mould of dimensions 25 mm*5 mm*2.5 mm and stored for 24 hours and 7 days in artificial saliva. The fracture toughness testing was done in a 3-point bend apparatus using Universal Testing Machine with a crosshead speed of 1 mm/min until failure. The mean fracture toughness values were compared using two-way ANOVA followed by post hoc (Bonferreni). The values at 24 hours and 7 days were compared using paired t-test.

**Results:**

At 24 hours and 7 days, Advanced Glass Hybrid GIC showed the highest fracture toughness values, while the lowest values were observed with conventional GIC. A statistically significant difference was seen between Advanced Glass Hybrid GIC and conventional GIC at 24 hours. Zirconium reinforced GIC showed intermediate values. In intra-group comparison, an increase in values was seen with all 3 materials at 7 days, however the difference was statistically insignificant.

**Conclusions:**

Advanced Glass Hybrid and Zirconium reinforced GIC showed better fracture toughness than the Conventional GIC. Fracture toughness of GICs was influenced by time.

## Resumen

**Objetivo:**

El propósito de este estudio fue evaluar y comparar la tenacidad a la fractura del cemento de ionómero de vidrio (CIV) híbrido de vidrio avanzado, el CIV reforzado con zirconio y el CIV convencional, mediante un ensayo de flexión de tres puntos.

**Materiales y métodos:**

Se formaron tres grupos en el presente estudio: Grupo 1 (CIV híbrido), Grupo 2 (CIV mejorado con zirconio) y Grupo 3 (CIV convencional). Los grupos se sometieron a ensayos a intervalos de 24 horas y 7 días, según su tiempo de almacenamiento en saliva artificial. Se prepararon muestras de cada material con un molde personalizado de 25 mm x 5 mm x 2,5 mm, y se almacenaron durante 24 horas y 7 días en saliva artificial. El ensayo de tenacidad a la fractura se realizó en un aparato de flexión de tres puntos utilizando una máquina de ensayos universal con una velocidad de cruceta de 1 mm/min hasta la rotura. Los valores medios de tenacidad a la fractura se compararon mediante un ANOVA de dos vías seguido de un análisis post hoc (Bonferreni). Los valores a las 24 horas y a los 7 días se compararon mediante una prueba T pareada.

**Resultados:**

A las 24 horas y a los 7 días, el CIV híbrido de vidrio avanzado mostró los valores más altos de tenacidad a la fractura, mientras que los valores más bajos se observaron con el CIV convencional. Se observó una diferencia estadísticamente significativa entre el CIV híbrido de vidrio avanzado y el CIV convencional a las 24 horas. El CIV reforzado con circonio mostró valores intermedios. En la comparación intragrupo, se observó un aumento en los valores con los tres materiales a los 7 días; sin embargo, la diferencia fue estadísticamente insignificante. Conclusiones: El CIV híbrido de vidrio avanzado y el CIV reforzado con circonio mostraron una mejor tenacidad a la fractura que el CIV convencional. La tenacidad a la fractura de los CIV se vio influenciada por el tiempo.

## INTRODUCTION

Dental caries is one of the most common chronic diseases seen in children around the world ^1^. According to World Health Organization, the global prevalence of primary dentition caries ranges between 60% and 90% and is fourth-most expensive chronic disease to treat ^2^. Primary teeth play an important role in development of children thus it is crucial to retain these teeth for as long as possible ^1^. Furthermore, pain and abscess may be seen in teeth with untreated dental caries. Caries represents a biological, social, and economical burden on individuals and health-care systems. Hence, it is important to intervene in the early stages by arresting carious process and sealing with a restorative material ^2^. It is desirable that the restoration placed in teeth should be maintained till their exfoliation. Dental amalgam was the most used restorative material for many years until concerns of toxic effects of mercury discontinued its use ^3^.

Currently, GIC as a restorative material is commonly used for primary teeth owing to its anticariogenic and biocompatible properties. GIC binds to the hard tissues of teeth through chemical bonding. Formation of ionic bond occurs between polyacrylic and carboxylic groups of the cement and hydroxyapatite crystals of enamel and dentin ^4^. This makes it an ideal restorative material for primary teeth. GIC shows low thermal expansion and high fluoride release which proves to be one of the most important features of GIC ^5^. These properties attribute to the low rate of secondary caries formation in GIC restorations. However, one important factor is the durability of the restoration.

Physical properties such as fracture toughness play an important role in determining the durability of the restoration. In literature, many authors have conducted research on fracture toughness of restorative materials. In a study by Kovarik et al. ^6^, it was mentioned that fracture toughness is an intrinsic property of a material and is the measure of a material's resistance to crack propagation. This property can be measured using various methods such as Compact tension method, the double torsion method, Chevron notch method but the most efficient and reliable test was 3-point bending test ^7^.

GIC despite having the advantages of chemical bonding and fluoride release, lacks the strength for its use as load bearing restoration in posterior teeth. Its use is limited due to low tensile strength, poor esthetics and low final hardness ^8^^,^^9^. Conventional GIC which was introduced in 1972, has certain disadvantages such as low wear resistance and inferior flexural strength and toughness. As per fracture toughness regarding conventional GICs, it is inferior when compared to resin modified GIC and composite resin ^10^. The wear rate of GICs is five times higher than amalgam and three times higher than composite-resin materials ^11^.

Due to such limitations of GIC, modifications of its composition were developed in order to improve the physical and mechanical properties. This includes addition of metal particles, changes in the glass particle size, water-activated dehydrated polyacid powder and aluminium-free glass ^12^.

One such modification was made by GC Corporation (Tokyo, Japan) which is known as GC Gold Label Hybrid. It is modified with advanced glass hybrid technology that combines two different types of Fluoro-Alumino-Silicate (FAS) glass and two different types of polyacrylic acid. The reaction between them causes high fluoride release by the restorative matrix. The addition of these components strengthens the restorative matrix and makes it highly resistant to acid. 

Another modification, zirconium reinforced GIC (Zirconomer Improved, Shofu Inc., Japan), was introduced to overcome the limitations of conventional GICs. It contains zirconium oxide, glass powder, tartaric acid, polyacrylic acid and deionized water as its liquid. Zirconomer Improved was introduced to match the strength and longevity of amalgam with fluoride release of glass ionomer without the hazardous effects of mercury. This variant of GIC is reinforced with nano-zirconia fillers, which impart higher mechanical properties to the restoration making it suitable for restoration of load bearing areas in patients with high caries risk ^13^^,^^14^.

As Conventional GICs have shown inferior fracture toughness in the literature ^10^^,^^15^, newer materials such as advanced glass hybrid GIC and zirconium reinforced GIC have been introduced with better mechanical and physical properties. Studies comparing fracture toughness of conventional GIC and zirconium reinforced GIC have been done previously ^16^. In literature, there is no information available on the fracture toughness of advanced glass hybrid GIC. Hence, the aim of this study was to evaluate and compare the fracture toughness of advanced glass hybrid GIC, zirconium reinforced GIC and conventional GIC using three-point bending test.

## MATERIAL AND METHODS

### Ethical Approval

This was an in-vitro experimental study designed according to CRIS guidelines ^17^. Ethical approval was obtained from the Institutional Review Board- Ethics Committee (IRB-EC) (TDC/EC/34/2022). 

### Sample Size Calculation

The restorative materials used in this study were three self-curing GICs. The type and composition of the materials are listed in Table 1. Sample size was determined from a study reported by Al-Angari et al. ^18^, through G* power software. Sample size was determined to be 6 per group which was rounded off to 10 per group. The total sample size was 60. 

**Table 1:** Specification of materials used

Material	Manufacturer	Type	Composition
GC Gold Label Hybrid	GC Corporation, Tokyo, Japan	Hybrid Glass Ionomer Cement	Acid-resistant Fluoro alumino silicate glass and high molecular weight polyacrylic acid
Zirconomer Improved	Shofu INC, Japan	Zirconia reinforced restorative glass ionomer	Powder: Alumino Fluoro silicate glass, Zirconium oxide, Tartaric acid Liquid: Polyacrylic acid, Deionized water
GC Gold Label High Strength Posterior Extra	GC Corporation, Tokyo, Japan	Conventional Glass Ionomer Cement	Powder: Fluoro alumino silicate glass, Polyacrylic acid powder. Liquid: Polyacrylic acid Polybasic carboxylic acid

### Preparation of sample

**Figure 1:**
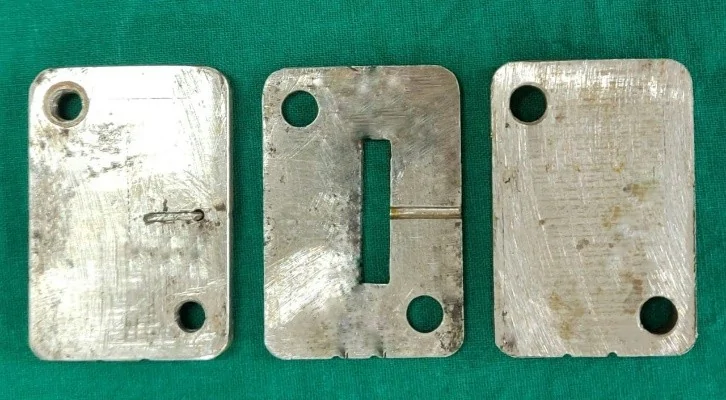
Customized metal mould

**Figure 2:**
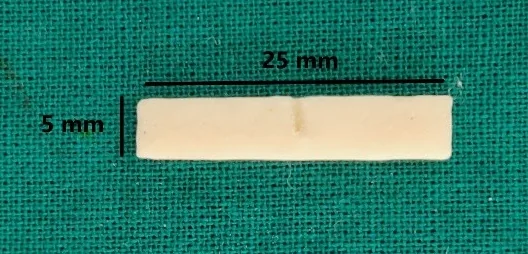
Glass Ionomer Cement Sample

A customized metal mould (Figure 1) was fabricated with the standardized dimensions (25 mm*5 mm*2.5 mm) in accordance with Single Edge Notch Beam (SENB) method. [3] A metal plate with a V-shaped edge was placed in the mould to create a V-shaped notch measuring 1 mm in the sample. For easy retrieval of the sample, separating medium was applied in the mould. Powder and liquid of GIC was manipulated using the manufacturer’s instructions and filled in the cavity of the mould. The samples were made by applying even pressure by compressing the restorative material from both sides using the plates of the metal mould. The samples were allowed to set for 10 minutes after which they were carefully retrieved from the mould and examined for any minor irregularities. If present, they were removed by carefully grinding with fine grit sand paper (Figure 2).

The samples were categorized in 3 groups (n = 20 in each group) of restorative material, which were further subdivided into two subgroups according to storage time in artificial saliva (n = 10 in each subgroup).

**Figure 3:**
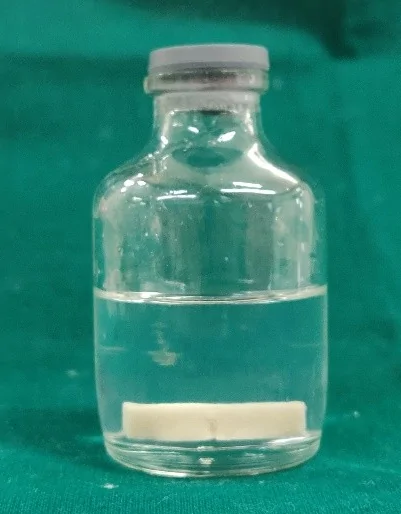
Storage of sample in artificial saliva

The samples were stored in artificial saliva for 24 hours and 7 days respectively before testing (Figure 3). The sample division in groups are given as follows:

In Group 1A and 1B, advanced glass hybrid GIC samples were stored for 24 hours and 7 days respectively. In Groups 2A and 2B, zirconium-reinforced GIC samples were stored for 24 hours and 7 days respectively. In Groups 3A and 3B, conventional GIC samples were stored for 24 hours and 7 days respectively.

### Fracture toughness analysis

**Figure 4:**
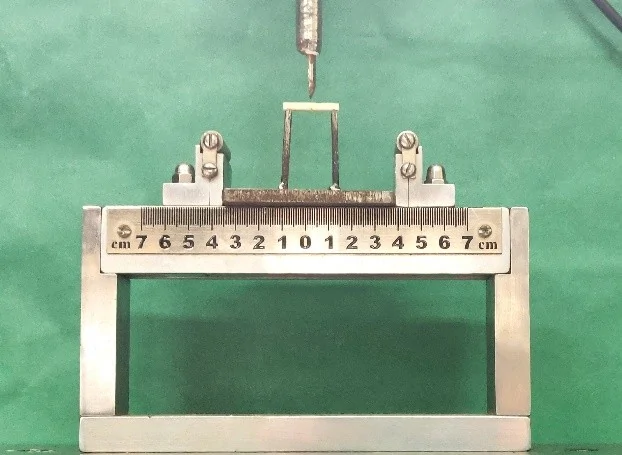
3-point bending test in Universal Testing Machine

The samples were placed in a Universal Testing Machine with the notch facing the tension side and fractured using 3-point bending test with a cross-head speed of 1 mm/minute (Figure 4). The notch depth (a), height of specimen (h) and width of specimen (w) were measured. The Span distance (S) was 20 mm. The value of F (Maximum force) at which the specimen fractured was noted and the value for fracture toughness (KIC) was calculated using the following formula:

KIC = 3 (a/W)1/2 (1.99- a/W (1-a/W) (2.15 - 3.93a/W + 2.7(a/W)2) FS

2(1+2a/W) (1-a/W)3/2 hW3/2

### Statistical analysis

The data collected was tabulated and subjected to statistical analysis using Statistical Package of Social Sciences software (IBM SPSS, version 17, Chicago, IL, USA). Statistical analysis was done using two-way ANOVA and *post hoc* test for intergroup and intragroup comparisons. The values at 24 hours and 7 days were compared using Paired t test. A p-value < 0.05 was considered statistically significant.

## RESULTS

The mean fracture toughness values of the materials tested are listed in Table 2. 

**Table 2:** GIC mean fracture toughness (MPa √m)

Material	24 hours			7 days	
	Mean	S.D.	Mean	S.D.
Hybrid GIC	0.292	(0.07)	0.298	(0.04)
Zirconomer Improved	0.257	(0.03)	0.287	(0.05)
Conventional GIC	0.229	(0.03)	0.259	(0.06)

The obtained results were compared using two-way ANOVA and post hoc test. Paired t test was used to compare the values at 24 hours and 7 days in all three groups. The mean fracture toughness value of conventional GIC at 24 hours and 7 days was the lowest among the materials tested which was 0.229 MPa m^1/2^ and 0.259 MPa m^1/2^ respectively. The values recorded at 24 hours for advanced glass hybrid GIC was 0.292 MPa m^1/2^ and 0.298 MPa m^1/2^ at 7 days which was the highest among the materials tested. Two-way ANOVA showed that the type of tested material shows a statistically significant difference on fracture toughness values (p = 0.018). However, the storage period and interaction between the type of material tested and storage period are statistically insignificant (p = 0.127) (p = 0.729) respectively (Table 3). Advanced glass hybrid GIC yielded significantly greater values than Conventional GIC (p < 0.05) at 24 hours while the difference between both materials at 7 days was statistically insignificant (Table 3, 4). On the other hand, zirconium reinforced GIC revealed intermediate fracture toughness values at both 24 hours and 7 days. 

**Table 3:** Results of two-way ANOVA for fracture toughness

Source of variation	Sum of squares	Df	Mean square	F	p value
Material	0.026	2	0.013	4.319	0.018**
Storage period	0.007	1	0.007	2.403	0.127
Material * Storage period	0.002	2	0.001	0.318	0.729

** significant value set at p<0.05

## DISCUSSION

Glass ionomer cements have been widely used as a restorative material for primary teeth ^3^. In permanent teeth, Composite resins are preferred over GIC for restoration in stress bearing areas due to higher strength and durability. GIC is preferred in primary teeth due to its anticariogenic property, fluoride release and most importantly, it is less technique sensitive and hydrophilic in nature which makes it a suitable restorative material for young patients ^19^. However, conventional GICs are not indicated for restoration of posterior teeth as they have low wear resistance and marginal integrity when compared to composite resins. A material's resistance to crack initiation and propagation is indicated by its fracture toughness, a distinctive factor that is important when evaluating survival of the restoration. In a study by Manhart ^20^, it was observed that an annual failure rate of 7.2% resulted from fractures in conventional GIC restorations, higher than amalgam (3.0%) or composite restorations (2.2%) in posterior teeth. This led to development of newer formulations of GIC with improved physical properties. This study used a three-point bending test to assess and compare the fracture toughness of newer modified GICs with conventional GIC.

Fractured restorations are a primary reason for failure and is a major factor in their success and longevity ^3^. This fracture may be the culmination of crack propagation in the restoration or due to heavy masticatory forces ^21^. Fracture toughness or the critical stress intensity is a mechanical property that describes the resistance of brittle materials to the catastrophic propagation of flaws under an applied stress ^22^. This test relates to chipping and bulk fracture of a restoration and may be an essential laboratory factor in determining the resistance of a restorative material to intraoral fracture ^23^.

In this study, the materials used were three self-curing Glass Ionomer Cements. The samples were prepared and stored in artificial saliva for 24 hours and 7 days. Fracture toughness evaluation was done at 24 hours, as it has been stated that this represents a time when restorative materials are usually first subjected to oral forces. For all the tested materials, the 7 days storage period resulted in higher mean fracture toughness value compared to 24 hours storage, however the intra group and inter group comparisons showed no significant difference. This is mainly due to the maturation of the GIC. It has been observed that the maturation process improves physical properties of GIC notably in the first 24 hours and continues for up to a year ^24^.

Different studies have established that the acid base reaction of GIC is primarily complete by 24 hours ^25^^,^^26^. Zainuddin et al. ^27^ reported that following acid-base reaction, the formation of a network and a hydrated silica gel phase occurs. This consists of either a pure silicate or a mixed silicate phosphate matrix that contributes to improvement in physical property with continued matrix maturation.

The SENB method used in the current study has been commonly used to determine the fracture toughness primarily because of its simplicity. Fracture toughness measurements are often performed using a three- or four-point bending device, both of which are commonly used in dental material research. The 3-point bending test has been shown to simulate the clinical situation in which a cusp of the opposing tooth exerts force on the restoration ^10^. The comparison between three-point and four-point bending tests highlights that there is not a large difference between them ^28^. Hence, three-point bending test was used in this study.

The two-way ANOVA compares the mean differences between groups that have been split on two independent variables. The primary purpose of a two-way ANOVA is to understand if there is an interaction between the two independent variables on the dependent variable. In this study, two-way ANOVA calculated the interaction between materials tested and the storage period on fracture toughness. Hence, this test was used for analyzing data in the present study.

The current study found that fracture toughness of Zirconium reinforced GIC was higher than conventional GIC. This was in accordance to results of the studies conducted by Vemagiri C et al. ^29^ and Chalissery ^13^ which showed that Zirconium reinforced GIC had better physical properties compared to Ketac molar. The inclusion of zirconium as a filler particle in the glass component of Zirconomer Improved makes it suitable for restoration in posterior teeth by improving the mechanical properties of the restoration. The presence of reinforcing particles in a material distributes the propagating force. This causes the crack to dissipate between particles and becomes unfavourable for crack to grow. This makes the restoration difficult to fracture. This can be an explanation for better fracture toughness observed with Zirconium reinforced as compared to Conventional GIC.

Advanced Glass Hybrid GIC consists of more acid resistant FAS glass and a higher molecular weight polyacrylic acid. The smart particle size distribution provides a stronger matrix and impart higher mechanical strength thus showing better fracture toughness as compared to other materials. Although no studies are available evaluating fracture toughness of Hybrid GIC. A recent study by Malhotra S et al. ^30^ compared only the compressive strength and flexural strength of Advanced Glass Hybrid GIC, conventional GIC and Resin Modified GIC. Another study by Agrawal ^31^ compared the shear bond strength of the materials used in the present study. Hence, present study will highlight the fracture toughness of Advanced glass hybrid GIC.

The results of the current study were similar to those reported by Lloyd and Butchart ^32^ who investigated the fracture toughness of conventional GIC, cermet and resin composites using a SENB specimen. The highest KIC values were observed with composite resin ranging from 1.0 to 1.38 MN · m23/2. The KIC values for the glass ionomer, cermet and alloy/glass-ionomer mix ranged from 0.21 to 0.48 MN · m23/2 which were comparable with the KIC values of this study.

A study done by Yamazaki et al. ^33^ tested the viscoelastic behavior and fracture toughness of six glass ionomer cements. In this study, three conventional (a-Silver, a-Fil, and Ketac-Molar) and three Resin modified GIC (Vitremer, Fuji II LC, and Photac-Fil Quick) were checked for fracture toughness. No significant difference was found among the materials tested although resin modified GIC displayed higher fracture toughness.

The current study included materials that have been traditionally used such as GC High Strength Posterior (conventional GIC) and included newer materials such as GC Hybrid GIC (Advanced Glass Hybrid) and Zirconomer Improved (Zirconium reinforced GIC) which have not been previously reported in the scientific literature for fracture toughness.

## CONCLUSION

Advanced Glass Hybrid GIC and Zirconium reinforced GIC showed better fracture toughness values at both 24 hours and 7 days. Conventional GIC showed the least fracture toughness values amongst the materials tested. It can be concluded that fracture toughness is associated with storage period as all materials showed increase in values after testing at 7 days.
